# Fluorescence Polarization Binding Assay for *Aspergillus fumigatus* Virulence Factor UDP-Galactopyranose Mutase

**DOI:** 10.4061/2011/513905

**Published:** 2011-08-21

**Authors:** Jun Qi, Michelle Oppenheimer, Pablo Sobrado

**Affiliations:** ^1^Department of Biochemistry, Virginia Tech, Blacksburg, VA 26061, USA; ^2^Enzyme Research and Drug Discovery Laboratory, Virginia Tech, Blacksburg, VA 26061, USA; ^3^Fralin Life Science Institute, Virginia Tech, Blacksburg, VA 26061, USA

## Abstract

*Aspergillus fumigatus* is an opportunistic human pathogenic fungus responsible for deadly lung infections in immunocompromised individuals. Galactofuranose (Gal*f*) residues are essential components of the cell wall and play an important role in *A. fumigatus* virulence. The flavoenzyme UDP-galactopyranose mutase (UGM) catalyzes the isomerization of UDP-galactopyranose to UDP-galactofuranose, the biosynthetic precursor of Gal*f*. Thus, inhibitors of UGM that block the biosynthesis of Gal*f* can lead to novel chemotherapeutics for treating *A. fumigatus*-related diseases. Here, we describe the synthesis of fluorescently labeled UDP analogs and the development of a fluorescence polarization (FP) binding assay for *A. fumigatus* UGM (A*f*UGM). High-affinity binding to A*f*UGM was only obtained with the chromophore TAMRA, linked to UDP by either 2 or 6 carbons with *K_d_* values of 2.6 ± 0.2 **μ**M and 3.0 ± 0.7 **μ**M, respectively. These values were *~*6 times lower than when UDP was linked to fluorescein. The FP assay was validated against several known ligands and displayed an excellent Z′ factor (0.79 ± 0.02) and good tolerance to dimethyl sulfoxide.

## 1. Introduction

*Aspergillus fumigatus* is an opportunistic human pathogen responsible for diseases such as allergic reactions and lung infections, including bronchopulmonary aspergillosis (ABPA) and invasive pulmonary aspergillosis (IPA) [1, 2]. This fungus is a significant health threat to immunocompromised patients, such as organ transplant recipients and people with AIDS or leukemia [3, 4]. It has been reported that IPA infections are typically accompanied by a mortality rate of 50–70% [5]. Thus, identification of novel and effective drug targets is essential in the fight against fungal infections. 

**Figure 1:**
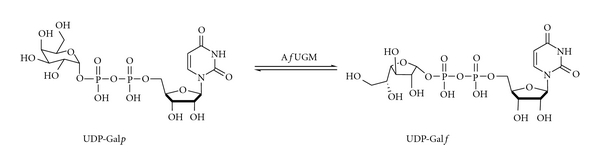
Reaction catalyzed by *Af*UGM.

Recently, the biosynthetic pathway of galactofuranose (Gal*f*), the 5-membered ring form of galactose, has been described in *A. fumigatus*. Gal*f* is a component of the cell wall of *A. fumigatus* and plays an important role in virulence [6–8]. In *A. fumigatus*, Gal*f* was first identified as a component of galactomannan by immunodetection in IPA patients [9]. Later, it was found that Gal*f* is also a major component of saccharide structures in membrane lipids and glycosyl phosphoinositol (GPI-)anchored lipophospholipids [10, 11]. UDP-galactopyranose mutase (UGM) is a flavoenzyme that catalyzes the conversion of UDP-galactopyranose (UDP-Gal*p*) to UDP-galactofuranose (UDP-Gal*f*, Figure 1), the biosynthetic precursor of Gal*f * [7, 12]. Deletion of the *A. fumigatus* UGM (*Af*UGM) gene results in mutant fungi with attenuated virulence, a decrease in cell wall thickness, and an increase in the sensitivity to antifungal agents [8, 13]. Moreover, Gal*f* is absent in humans [12]. Thus, inhibitors of *Af*UGM that block the biosynthesis of Gal*f* represent attractive drug targets for the identification of novel therapeutics against *A. fumigatus*.

**Figure 2:**
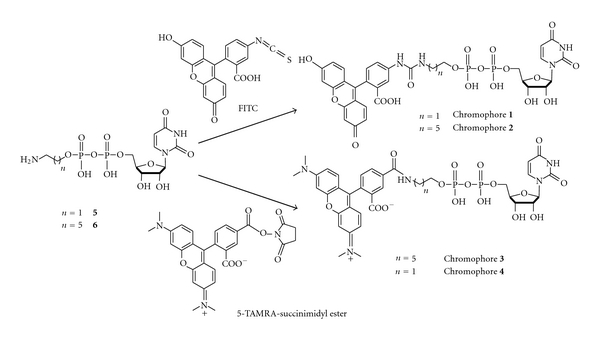
Synthetic scheme of the chromophores used as ligands to *Af*UGM for application in FP assays.

Here, we describe the development of a fluorescence polarization (FP) binding assay to identify specific *Af*UGM inhibitors. Four fluorescently labeled UDP derivatives including two known UDP-fluorescein analogs (**1** and **2**, Figure 2) and two novel UDP-TAMRA analogs (**3** and **4**, Figure 2) were synthesized to be used as fluorescent probes in the FP assay. Their concentrations were optimized to obtain a stable FP signal with minimal standard deviation, and their *K*_*d*_ values were determined by measuring the anisotropy changes as a function of *Af*UGM concentration. We found that the UDP-TAMRA analogs bind to *Af*UGM 6-fold tighter than the UDP-fluorescein analogs, suggesting that UDP-TAMRA analogs are better fluorescent probes for this enzyme. UDP-TAMRA probes could be competed out by UDP, a known ligand of UGMs, and the *K*_*d*_ value of UDP was in good agreement with the value determined previously in a fluorescence assay [7]. Furthermore, the FP assay was validated using several known ligands and displayed an excellent Z′ factor (0.79 ± 0.02) and good tolerance to DMSO. Therefore, this fast convenient one-step FP assay is suitable for a high-throughput screening to identify *Af*UGM inhibitors. 

## 2. Materials and Methods

### 2.1. Materials

All chemicals were obtained from commercial sources and were used without further purification. Anhydrous reactions were performed under argon. All solvents were either reagent grade or HPLC grade. NMR spectral data were obtained using a JEOL Eclipse spectrometer at 500 MHz, or a Varian Inova spectrometer at 400 MHz. Chemical shifts were reported as **δ**-values relative to known solvent residue peaks. High-resolution mass spectra (HRMS) were obtained in the Mass Spec Incubator, Department of Biochemistry, Virginia Tech. High-performance liquid chromatography (HPLC) was performed on a C18 reverse phase column (Phenomenex Luna C18 column, 250 × 21.20 mm, 5 microns) using water and acetonitrile as the elution solvents. All compounds were more than 95% pure as judged by HPLC and ^1^H NMR.

### 2.2. Protein Expression and Purification

*Af*UGM and *Mt*UGM were expressed and purified with the same protocol as described by Oppenheimer et al. [7]. A large quantity of highly pure *Af*UGM was obtained, which was confirmed by UV-visible spectrophotometry and SDS-PAGE (see Figure  S1 Supplementary Material available online at doi:10.4061/2011/513905).

### 2.3. Synthesis of UDP-Fluorescein Chromophore 1 and 2

The synthesis of chromophore **1** was accomplished by reacting 4 mg of compound **5**, which was synthesized following a previously published procedure [15], with 6 mg of fluorescein-5-isothiocyanate (FITC) in 0.1 M pH 9.0 NaHCO_3_ buffer (50 *μ*L) and DMF (100 *μ*L) (Figure 2). After stirring at room temperature for 2 hours, the yellow solution was concentrated and loaded onto a preparative silica gel TLC plate. The isolated crude product was dissolved in water, injected onto reverse-phase HPLC (Phenomenex Luna C18 column, 250 × 21.20 mm, 5 microns), and purified at a flow rate of 5.0 mL/min with linear gradient elution of 5% to 95% acetonitrile in H_2_O over 20 min to afford chromophore **1** (4 mg, 52%). ^1^H NMR (500 MHz, 6 : 1 D_2_O: *d*_7_-DMF): **δ** 7.96 (d, *J* = 8.2, 1H), 7.78 (s, 1H), 7.70 (d, *J* = 8.1, 1H), 7.30 (dd, *J* = 8.2, 1.5, 1H), 7.27-7.27 (m, 2H) (t, *J* = 8.7, 2H), 6.65–6.61 (m, 2H), 6.61–6.58 (m, 2H), 5.91 (s, 1H), 5.91 (s, 1H), 4.36–4.30 (m, 2H), 4.24–4.21 (m, 3H), 4.19–4.16 (m, 2H), 3.88 (s, 2H); HRMS (MALDI) calcd for C_32_H_29_N_4_O_17_P_2_S (M-H)^−^: 835.0729, found 835.0759 (Figure  S2).

Chromophore **2** (7.1 mg, 55%) was synthesized from the reaction of compound **6** and FITC by the same procedure as above (Figure 2) and was purified by preparative TLC and reverse-phase HPLC (Phenomenex Luna C18 column, 250 × 21.20 mm, 5 micron). ^1^H NMR (500 MHz, 6 : 1 D_2_O: *d*_7_-DMF): **δ** 8.00 (d, *J* = 8.0, 1H), 7.73 (s, 1H), 7.61 (d, *J* = 8.3, 1H), 7.31 (d, *J* = 8.3, 1H), 7.27-7.27 (m, 2H), 6.66–6.60 (m, 4H), 5.96 (s, 1H), 4.38–4.34 (m, 2H), 4.27–4.23 (m, 2H), 4.23–4.18 (m, 2H), 3.97–3.92 (m, 2H), 3.58 (s, 1H), 1.66–1.61 (m, 4H), 1.42–1.36 (m, 4H); HRMS (MALDI) calcd for C_36_H_37_N_4_O_17_P_2_S (M-H)^−^: 891.1350, found 891.1348 (Figure  S3).

### 2.4. Synthesis of UDP-TAMRA Chromophore 3 and 4

The synthesis of chromophore **3** was accomplished by a reaction of 4 mg of compound **6**, which was synthesized following a previously published procedure [15], with 0.8 mg of 5-carboxytetramethylrhodamine, succinimidyl ester (5-TAMRA, SE) in 0.1 M pH 8.3 NaHCO_3_ buffer (50 *μ*L) and DMF (50 *μ*L) (Figure 2). After stirring at room temperature for 2 hours, the pink solution was concentrated and loaded onto a preparative TLC plate. The isolated crude product was dissolved in water, injected onto reverse-phase HPLC (Phenomenex Luna C18 column, 250 × 21.20 mm, 5 microns), and purified at a flow rate of 5.0 mL/min with linear gradient elution of 5% to 95% acetonitrile in H_2_O over 20 min to afford chromophore **3** (1.1 mg, 80%). ^1^H NMR (500 MHz, D_2_O) **δ** 8.22 (s, 1H), 8.08 (d, *J* = 7.7, 1H), 7.89 (d, *J* = 7.3, 1H), 7.60 (d, *J* = 8.8, 1H), 7.28–7.22 (m, 1H), 6.91–6.88 (m, 2H), 6.61 (s, 1H), 6.59 (s, 1H), 5.89–5.83 (m, 2H), 4.35–4.31 (m, 1H), 4.30–4.26 (m, 1H), 4.18–4.15 (m, 3H), 4.00 (dd, *J* = 13.3, 6.4, 2H), 3.49 (t, *J* = 6.8, 2H), 3.19 (s, 3H), 3.18 (s, 3H), 1.75 – 1.67 (m, 4H), 1.51–1.45 (m, 4H), 1.34 (s, 3H), 1.32 (s, 3H); HRMS (MALDI) calcd for C_40_H_46_N_5_O_16_P_2_ (M-H)^−^: 914.2415, found 914.2431 (Figure  S4). The above synthetic approach was also used to synthesize and purify chromophore **4** (1.5 mg, 77%). HRMS (MALDI) calcd for C_36_H_38_N_5_O_16_P_2_ (M-H)^−^: 858.1789, found 858.1851 (Figure S5).

### 2.5. Optimization of Chromophore Concentration

Solutions containing various concentrations of chromophore in 0.05 M sodium phosphate buffer (pH 7.0) were added to 12 wells in a 96-well half area black bottom plate (Corning) with final volumes of 25 *μ*L. FP was analyzed by a SpectraMax M5 plate reader (Molecular Devices). The parallel fluorescence emission (F_=_) and perpendicular fluorescence emission (F_⊥_) at 524 nm (for compounds **1** and **2**, excitation at 492 nm) or at 584 nm (for compounds **3** and **4**, excitation at 544 nm) were measured by a SpectraMax M5 plate reader (Molecular Devices), and the anisotropy (*r*) was calculated using (1), the minimal concentration at which stable FP signals with minimal standard deviations were chosen as the optimal concentration for the chromophore.

(1) $$\begin{array}{cc} r & =\frac{F_{=}-G\cdot F_{⊥}}{F_{=}+2G\cdot F_{⊥}} \end{array}$$

(2) $$\begin{array}{cc} y & =m_{1}+\left(m_{2}-m_{1}\right)\\ & \;\times \frac{\left(x+C_{t}+m_{3}\right)-\sqrt{{\left(x+C_{t}+m_{3}\right)}^{2}-4xC_{t}}}{2C_{t}} \end{array}$$

### 2.6. FP Binding Assay to Determine the Chromophore Binding Affinities

Solutions containing serially diluted *Af*UGM and 15 nM of chromophore in 0.05 M sodium phosphate buffer (pH 7.0) were incubated at room temperature for 5 minutes. Each experiment was done in triplicate in a 96-well black bottom plate at final volumes of 25 *μ*L. Fluorescence anisotropy was measured as indicated above, and the *K*_*d*_ values were obtained by fitting the anisotropy data to (2), where *m*_1_ and *m*_2_ are the minimum and maximum anisotropy values, respectively; *m*_3_ is the *K*_*d*_ value, and the total concentration of UDP-chromophore is represented by *C*_*t*_.

### 2.7. Determination of the Assay *Z*′ Factor

Solutions containing 2 *μ*M of *Af*UGM and 15 nM of chromophore **3** in the absence (negative control) and presence (positive control) of 300 *μ*M of UDP were incubated at room temperature for 5 minutes. Each solution was added to octuplicate wells in a 96-well half area black bottom plate with final volumes of 25 *μ*L. The Z′ factors were calculated using (3), where *μ*_−_ represents the mean anisotropy value of the negative control, and *μ*_+_ is the mean anisotropy value of the positive control; *σ*_−_ represents the standard deviation of the negative control, and *σ*_+_ is the standard deviation of the positive control. A Z′ factor of 0.79 ± 0.02 was obtained for chromophore **3**.

(3) $$Z^{\prime }=1-\frac{3\left(\sigma_{-}+\sigma_{+}\right)}{\mu_{-}-\mu_{+}}$$

### 2.8. Optimization of AfUGM Concentration

To determine the optimal concentration of *Af*UGM in the FP assay, solutions containing 15 nM of chromophore **3** and *Af*UGM at various concentrations in the absence (negative control) and presence (positive control) of 300 *μ*M of UDP were incubated at room temperature for 5 minutes. Each was added to octuplicate wells at a final volume of 25 *μ*L. FP was analyzed as indicated previously, and Z′ factors were calculated from (3).

### 2.9. Competitive Binding Experiments Using FP Inhibition Assay

Solutions (25 *μ*L) containing 2 *μ*M of *Af*UGM and 15 nM of chromophore **3** in 0.05 M sodium phosphate buffer (pH 7.0) were mixed with various concentrations of UDP, UDP-Gal*p*, **7**, or **8 **(Figure 8), and the reactions incubated at room temperature for 5 minutes. Each solution was done in triplicate. Anisotropy values were measured and the IC_50_ values obtained by fitting the data to (4), where *m*_1_ and *m*_2_ are the minimum and maximum anisotropy, respectively; *m*_3_ is the slope, and *m_4_* is the IC_50_. The *K*_*d*_ values were obtained using (5), where *K*_*i*_ is the binding affinity of chromophore **3** on *Af*UGM (2.6 ± 0.6 *μ*M), and I is the concentration of the chromophore (15 nM).

(4) $$y=m_{1}+\frac{\left(m_{2}-m_{1}\right)x^{m_{3}}}{m_{4}^{m_{3}}+x^{m_{3}}}$$

(5) $$K_{d}=\frac{IC_{50}}{1+\left(I/K_{i}\right)}$$

### 2.10. AfUGM Activity Assay

The *Af*UGM activity assay was performed by monitoring the formation of UDP-Gal*p* from UDP-Gal*f* by HPLC. A 20 *μ*L reaction containing 20 mM dithiothreitol, 0.5 mM UDP-Gal*f* in 25 mM HEPES, 125 mM NaCl buffer, pH 7.5 in the absence of **7** or **8** was initiated by the addition of *Af*UGM at a final concentration of 50 nM. After incubation at 37°C for 10 min, the reaction was quenched by heat denaturation (95°C for 5 min) in a DNA engine thermocycler (BioRad, Hercules, Calif, USA). The same reaction was also performed in the presence of **7** (500 *μ*M) or **8** (50 *μ*M). The suspension was centrifuged and the supernatant was injected onto a CarboPac PA100 (Dionex) anion-exchange column. The sample was eluted isocratically with 75 mM KH_2_PO_4_ (pH 4.5), and the absorbance at 262 nm was monitored to identify fractions of substrate and product. The substrate UDP-Gal*f* was eluted at 36.5 min, and the product UDP-Gal*p* was eluted at 28.3 min. The inhibition of *Af*UGM activity was indicated by the extent of conversion of UDP-Gal*f* to UDP-Gal*p.*

### 2.11. Tolerance to DMSO

To determine the tolerance of the assay to DMSO, solutions containing 2 *μ*M of *Af*UGM, 15 nM of chromophore **3**, and DMSO at various concentrations in the absence (negative control) and presence (positive control) of 300 *μ*M of UDP were incubated at room temperature for 5 minutes. Fluorescence anisotropy values and Z′ factors were calculated as indicated previously.

## 3. Results and Discussion

### 3.1. Assay Design and Optimization

**Figure 3:**
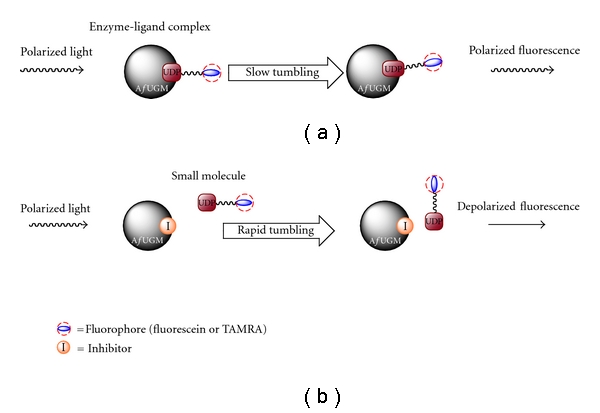
FP assay design. (a) Binding of the FP probe to *Af*UGM leads to polarized fluorescence. (b) Displacement of the FP probe from *Af*UGM by inhibitor results in depolarized fluorescence.

In this study, we report the development of an FP assay that can be used in a high-throughput format for the identification of inhibitors of *Af*UGM, which we believe will lead to the development of new therapeutics against *A. fumigatus-*related diseases. The FP assay was designed as shown in Figure 3. If the UDP fluorescent probe binds to *Af*UGM and is excited with plane-polarized light, the resulting enzyme-ligand complex tumbles slowly in solution, and thus, the fluorescence emission remains polarized (Figure 3(a)). Otherwise, the emission will be depolarized as the free chromophore will rotate rapidly. The change in the rotational motion between the bound and free chromophore can be used as a signal for detection of the binding of small molecules to the active site of *Af*UGM because, as the small molecule replaces the bound fluorescent probe, the free probe will rapidly rotate increasing the amount of depolarized fluorescence (Figure 3(b)). 

An essential component of an FP assay is a fluorescent probe that specifically binds to the enzyme or protein of interest. To design the fluorescent probe, we reasoned that the incorporation of the UDP moiety into the structure would target binding to the *Af*UGM active site since it is a major part of the UGM substrate. The fluorophore we first selected was fluorescein because UDP-fluorescein derivatives have been found to bind to prokaryotic UGMs from *Klebsiella pneumoniae* and *Mycobacterium tuberculosis* [15]. To minimize the steric hindrance of fluorescein with *Af*UGM binding site residues, UDP and fluorescein were connected with alkyl linkers of different lengths, which resulted in two UDP-fluorescein analogs (**1** and **2**, Figure 2). We also designed a UDP bound to the chromophore, commercially known as TAMRA (Figure 2). This chromophore offers several advantages over fluorescein. First, TAMRA is more resistant to photobleaching compared to fluorescein [16]. Second, the fluorescence emission of TAMRA does not overlap with that of the flavin cofactor in *Af*UGM. Fluorescein is typically excited at 494 nm and emits at 520 nm, which significantly overlaps with the absorbance and fluorescence emission of the flavin. In contrast, TAMRA's absorbance and fluorescence maxima is at 546 nm and 580 nm, respectively [16]. This is significantly different from the flavin absorbance/emission properties and improves signal-to-noise ratio. Finally, in comparison with fluorescein, TAMRA has one extra positive charge, which we believe increases the interaction between TAMRA and flavin and helps improve binding of the probe to *Af*UGM. Alkyl linkers of different lengths were also included to minimize the steric interaction of TAMRA with the binding site residues, giving two novel UDP-TAMRA analogs (**3** and **4**, Figure 2).

**Figure 4:**
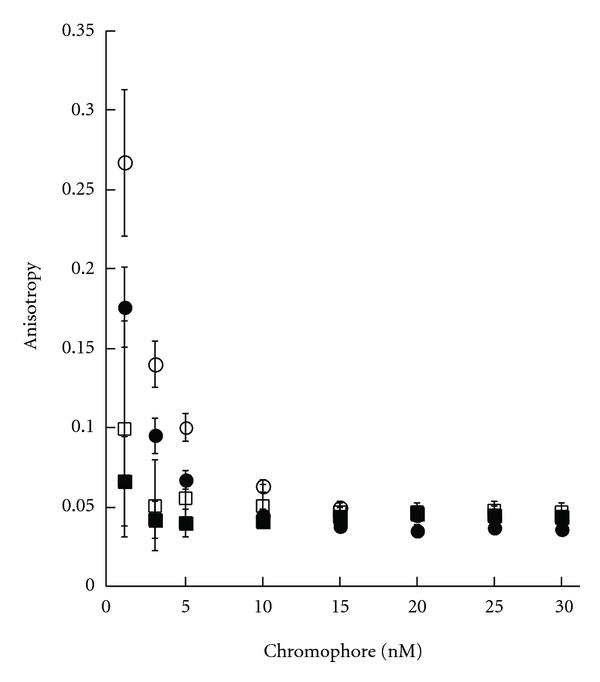
Determination of optimal concentration of fluorescent probe for FP binding assay. Conditions are described in Material and Methods sections. Chromophore **1** (○), **2 **(*⬤*) (excitation at 492 nm and emission at 524 nm), **3 **(■), and **4 **(□) (excitation at 544 nm and emission at 584 nm).

In order to increase the signal-to-noise ratio, stable FP values are necessary. Therefore, we varied the concentration of UDP-chromophores to determine the optimal concentration (Figure 4). Stable FP values with minimal standard deviation were obtained at concentrations higher than 15 nM. Therefore, we chose the 15 nM UDP-chromophore as the minimal concentration to use for further characterization.

### 3.2. AfUGM Specific UDP-Chromophore for HTS Assay Application

**Figure 5:**
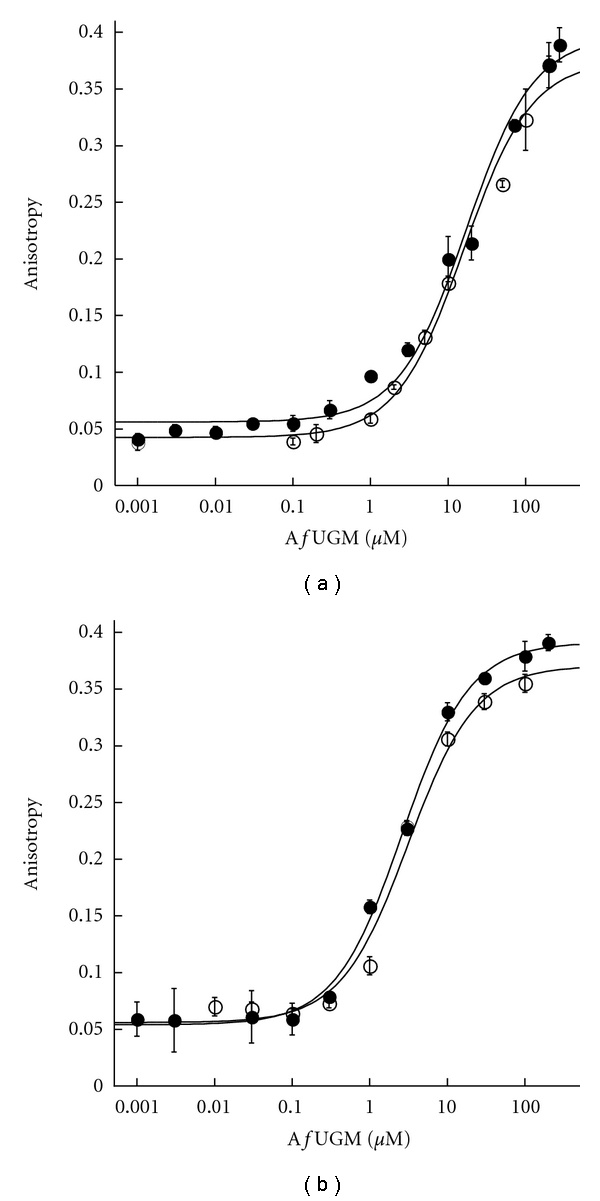
FP binding assay to determine *K*_*d*_ of the chromophores. (a) Chromophores **1** (○) and **2** (*⬤*) (excitation at 492 nm and emission at 524 nm). (b) Chromophores **3** (*⬤*) and **4** (○) (excitation at 544 nm and emission at 584 nm).

Binding of the UDP-chromophore to *Af*UGM was determined by varying the concentration of the enzyme at a constant concentration of the UDP-chromophores (15 nM) (Figure 5). Binding assays with the UDP-fluorescein probes (chromophores **1** and **2**) show that these ligands bind weakly to *Af*UGM, with *K*_*d*_ values of ~15 *μ*M (Figure 5(a)). This relatively low affinity impedes the utilization of these chromophores for a high-throughput FP binding assay, as it will require high quantities of enzyme. Interestingly, we tested the binding of these chromophores to bacterial UGM from *M. tuberculosis,* and the *K*_*d*_ value of chromophore **2** was 0.10 ± 0.01 *μ*M, consistent with previously published values (Table 1) [15]. This tighter binding suggests differences in the active-site architecture between the prokaryotic and *A. fumigatus *UGM enzymes. This is also consistent with our recent report on binding assays monitoring flavin fluorescence that showed that *Af*UGM binds UDP-glucose 5 times tighter than *K. pneumoniae* UGM. Similarly, binding of UDP-Gal*p *to *Af*UGM was not detected although UDP-Gal*p* binds to the bacterial enzyme with a *K*_*d*_ value of 220 *μ*M [7, 17]. These differences in ligand binding might originate from the low amino acid identity between the bacterial and eukaryotic UGMs (<18%). Furthermore, we have shown that the quaternary structure between these enzymes is not conserved as the bacterial enzymes have been shown to function as homodimers, while *Af*UGM functions as a homotetramer [7]. 

With the UDP-TAMRA analogs (chromophores **3** and **4**), the binding to *Af*UGM was ~6 times better than with the UDP-fluorescein analogs, and significant anisotropy change was measured (Figure 5(b)). Interestingly, the length of the linker had little or no effect on the binding affinities (Table 1), suggesting that with *Af*UGM the interaction between the chromophore and some components of the active site or perhaps directly with the flavin cofactor play a major role in binding. Compound **3** and **4** bound to *Mt*UGM with similar affinities as chromophores **1** and **2**, respectively. In contrast to *Af*UGM, in the bacterial enzymes, the length of the linker plays a major role in binding with longer linkers increasing the affinity, further demonstrating that the active-site architecture varied among the UGM enzymes. We selected **3** as the FP probe for further characterization of the binding assay.

### 3.3. Determination of Competitive Binding Using FP Assay

**Figure 6:**
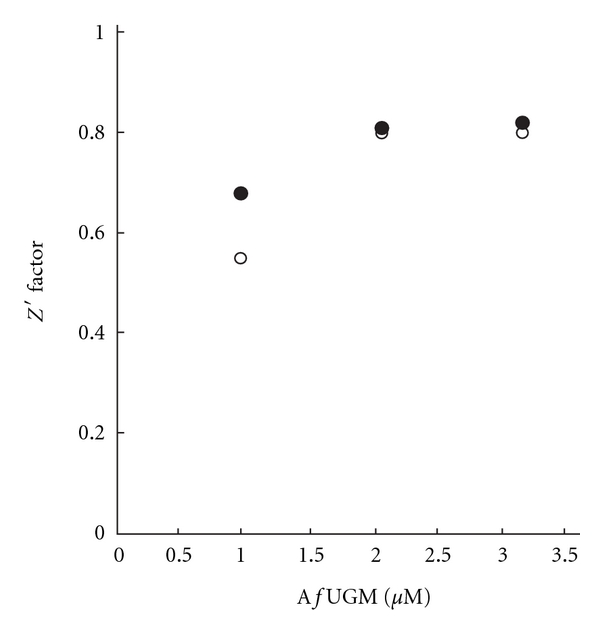
Determination of optimal *Af*UGM concentration to use in the FP assay with chromophore **3** (*⬤*) and chromophore **4 **(○).

FP competitive inhibition binding assay was conducted to confirm that the FP probes bind to the active site on *Af*UGM. First, the Z′ factor as a function of *Af*UGM was determined to establish the proper enzyme concentration to be used in the assay. The Z′ factor is a statistical parameter that reports on the quality of the assay [18]. As shown in Figure 6, the FP assay exhibits excellent quality at an *Af*UGM concentration higher than 2 *μ*M with a Z′ factor above 0.8. The minimum value (2 *μ*M) in this range was selected as the optimal assay concentration. 

**Figure 7:**
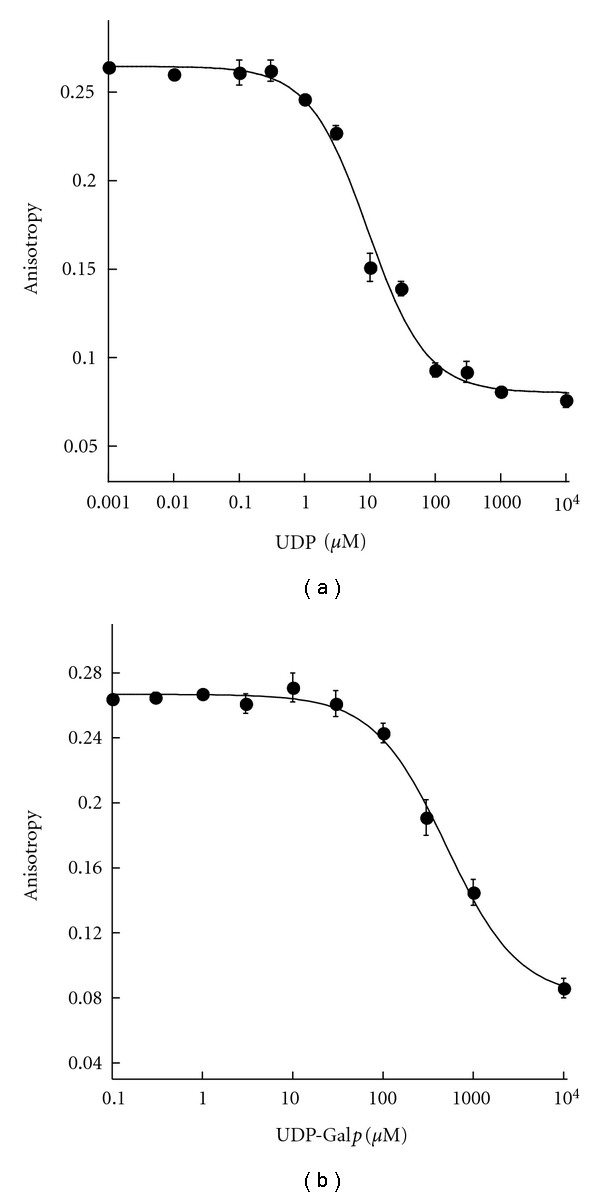
FP competitive binding assay with UDP (a) and UDP-Gal*p* (b).

The *K*_*d*_ for UDP was determined using the FP assay by titrating *Af*UGM with serial dilutions of UDP. A value of 9.0 ± 1.7 *μ*M was obtained, which is in good agreement with the *K*_*d*_ (33 ± 9 *μ*M) previously determined by directly monitoring the flavin fluorescence (Figure 7(a)) [7]. 

UDP-Gal*p* was the second ligand tested in the FP inhibition assay, and a *K*_*d*_ value of 495 ± 66 *μ*M was calculated (Figure 7(b)), indicating that UDP-Gal*p* is a poor ligand for *Af*UGM, which agrees well with the observation previously reported by Oppenheimer et al. [7]. 

**Figure 8:**
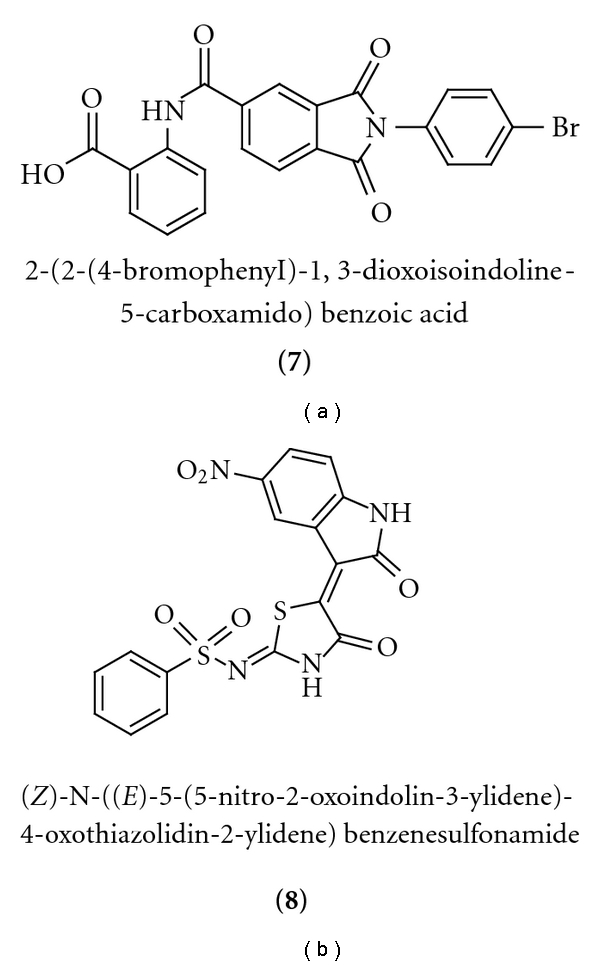
Structures of known inhibitors of bacterial UGM [14].

**Figure 9:**
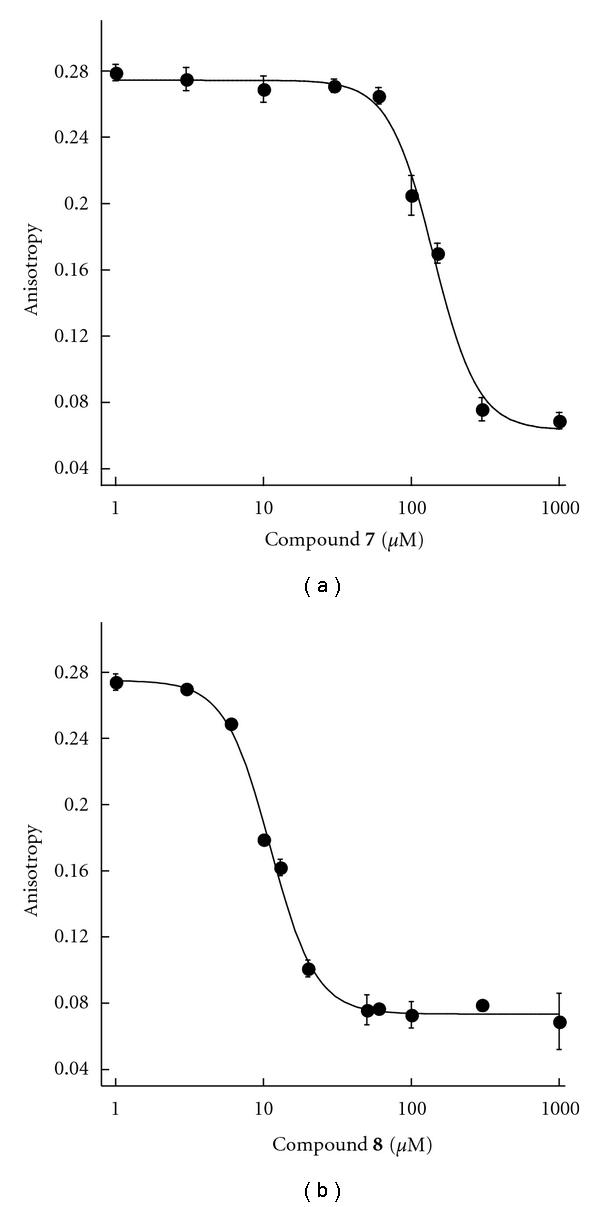
FP inhibition assay with compounds **7** (a) and **8** (b).

**Figure 10:**
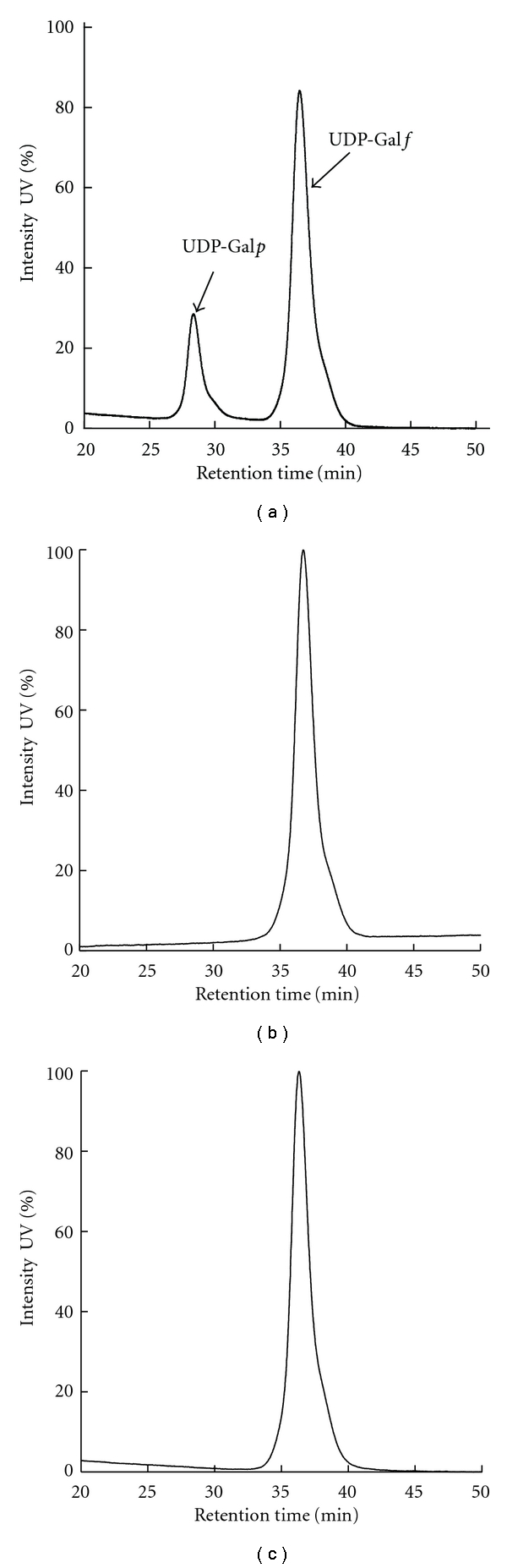
*Af*UGM activity assay. The HPLC chromatograms at 262 nm are shown. (a) *Af*UGM activity in the absence of inhibitor. (b) In the presence of **7 **(500 *μ*M). (c) In the presence of **8** (50 *μ*M).

Recently, a series of prokaryotic UGM inhibitors were identified from chemical libraries by high-throughput screening (HTS) [14]. In our FP assay, we tested two of the best prokaryotic UGM inhibitors, compound **7** and compound **8** (Figure 8). Interestingly, they behaved differently on *Af*UGM. Compound **7** turned out to be a poor ligand for *Af*UGM with a *K*_*d*_ of 140 ± 9 *μ*M (Figure 9(a)). In contrast, compound **8** exhibits much better binding to *Af*UGM (Figure 9(b)), and its *K*_*d*_ was found to be 11 ± 0.4 *μ*M (Table 2). We also tested these two compounds in a secondary assay, directly monitoring the activity of *Af*UGM to see if these molecules function as inhibitors. The HPLC chromatograms (Figure 10) indicated that both of the compounds inhibit the activity of *Af*UGM. These results confirm that the FP assay identifies ligands that bind to the active site of *Af*UGM and that these molecules inhibit the activity of the enzyme in a secondary assay that directly measures product formation.

### 3.4. FP Assay Quality

**Figure 11:**
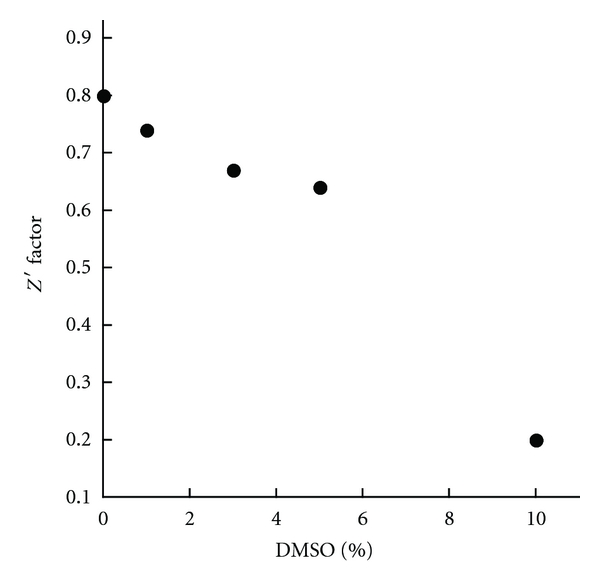
Tolerance to DMSO.

The Z′ factor value using chromophore **3** was calculated to be 0.79 ± 0.02. An assay with a Z′ factor greater than 0.5 is considered a good assay; therefore, our FP assay is suitable for HTS (Figure 6). We also estimated the tolerance of the FP assay to DMSO by calculating the Z′ factors at various DMSO concentrations, because a majority of compounds in HTS libraries are dissolved in DMSO. The Z′ factors were plotted against DMSO concentrations to generate a DMSO calibration curve (Figure 11), and our assay maintains excellent quality with DMSO concentration up to 5% (v/v). 

## 4. Conclusion

In conclusion, four fluorescently labeled UDP derivatives (**1**–**4**) were synthesized and tested for binding to *Af*UGM. Different from the bacterial UGM, the nature of the chromophore enhanced binding to AfUGM while the length of the linkers did not. UDP-TAMRA analogs (**3** and **4**) bind to *Af*UGM with high affinities. Binding of chromophore **3** to the active site of *Af*UGM was demonstrated by a competition experiment using UDP and UDP-Galp. Furthermore, binding of known inhibitors of bacterial UGM was tested against *Af*UGM, and it was found that these compounds bound *Af*UGM, however, with lower affinities. Inhibition of *Af*UGM, measuring product formation by HPLC, was demonstrated with compounds **7** and **8**. A Z′ factor of 0.79 was calculated, and the assay was shown to exhibit good tolerance to DMSO. We expect that the FP assay described here will allow fast identification of *Af*UGM inhibitors from chemical libraries. We believe that inhibitors of *Af*UGM that block the biosynthesis of Gal*f* could lead to novel therapeutics against *A. fumigatus-*related diseases.

## Supplementary Material

The Supporting Material includes the UV-visible spectrum and SDS-PAGE of *Af*UGM, and ^1^H NMR and High-resolution MS spectra of the four UDP chromophores. UV-visible spectrum was obtained on Agilent photospectrometer. NMR spectral data were obtained using a JEOL Eclipse spectrometer at 500 MHz, or a Varian Inova spectrometer at 400 MHz. Chemical shifts were reported as *σ*-values relative to known solvent residue peaks. High-resolution mass spectra (HRMS) were obtained in the Mass Spec Incubator of Department of Biochemistry at Virginia Tech.

## Figures and Tables

**Table 1:** *K*_*d*_ values of UDP-fluorescent probes.

Chromophore	*K*_*d*_ for *Af*UGM (*μ*M)	*K*_*d*_ for *Mt*UGM (*μ*M)
**1**	17 ± 3	>30
**2**	16 ± 3	0.10 ± 0.01
**3**	2.6 ± 0.2	0.73 ± 0.07
**4**	3.0 ± 0.7	>30

**Table 2:** *K*_*d*_ values of UGM ligands.

Ligand	*K*_*d*_ for *Af*UGM (*μ*M)	*K*_*d*_ for *Mt*UGM (*μ*M)
UDP	9.0 ± 1.7	15 ± 2
UDP-Gal*p *	495 ± 66	563 ± 75
**7**	140 ± 9	21 ± 1
**8**	11 ± 0.4	25 ± 2
