# Supplementary material for: Fluorescence Polarization Binding Assay for Aspergillus fumigatus Virulence Factor UDP-Galactopyranose Mutase
Source: Enzyme Res. 2011 Aug 21;2011:513905. doi: 10.4061/2011/513905 (PMC3159312; doi:10.4061/2011/513905)

Binding assay for UDP-galactopyranose mutase from *Aspergillus fumigatus*

Jun Qi[1,2], Michelle Oppenheimer[1,2], Pablo Sobrado[1,2,3*]

[1]Department of Biochemistry,[2]Enzyme Research and Drug Discovery Laboratory, [3]Fralin Life Science Institute, Virginia Tech, Blacksburg, VA 26061

psobrado@vt.edu

Supplementary data

Figure S1. UV-visible spectrum and SDS-PAGE of *Af*UGM

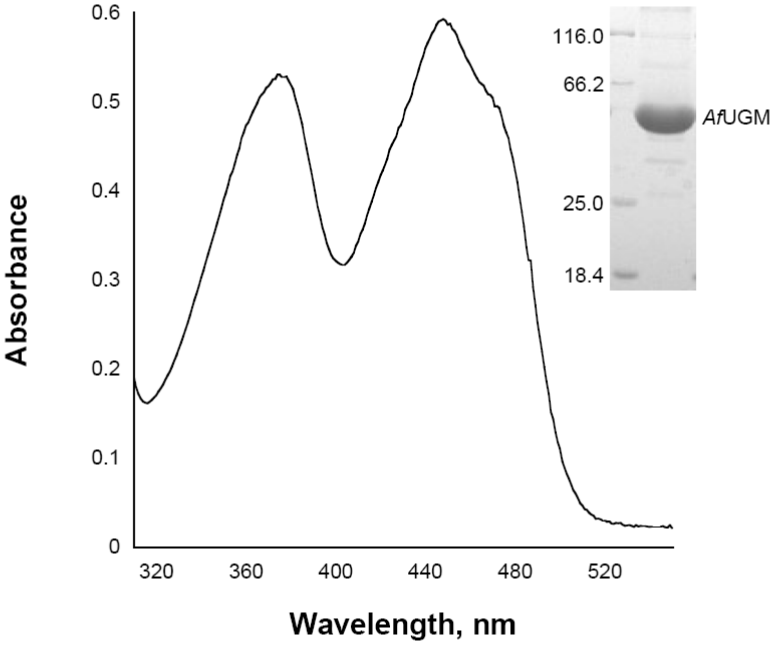

Figure S2. ^{1}H NMR spectrum and high-resolution mass spectrum of chromophore **1**

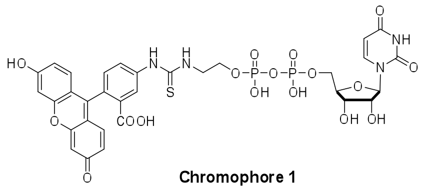

Chromophore 1

$C_{32}H_{29}N_4O_{17}P_2S$ $(M-H)^-$: calcd 835.0729, found 835.0759

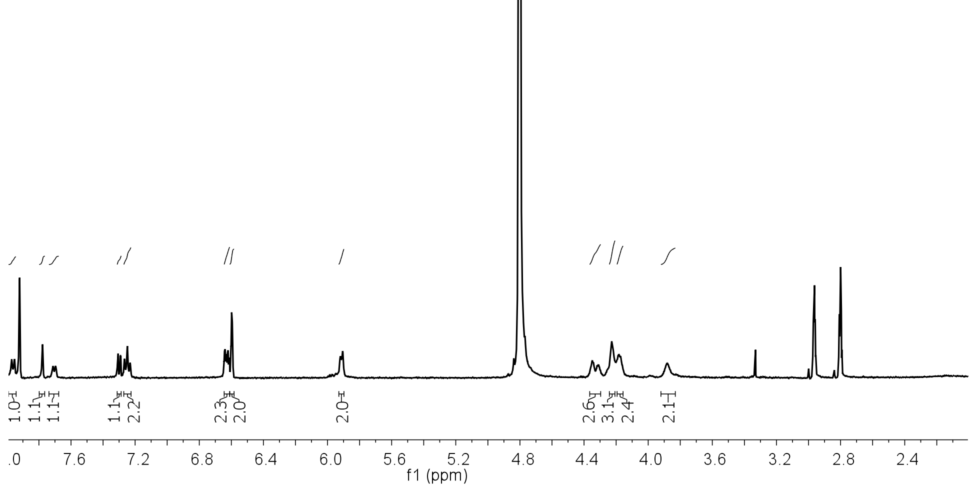

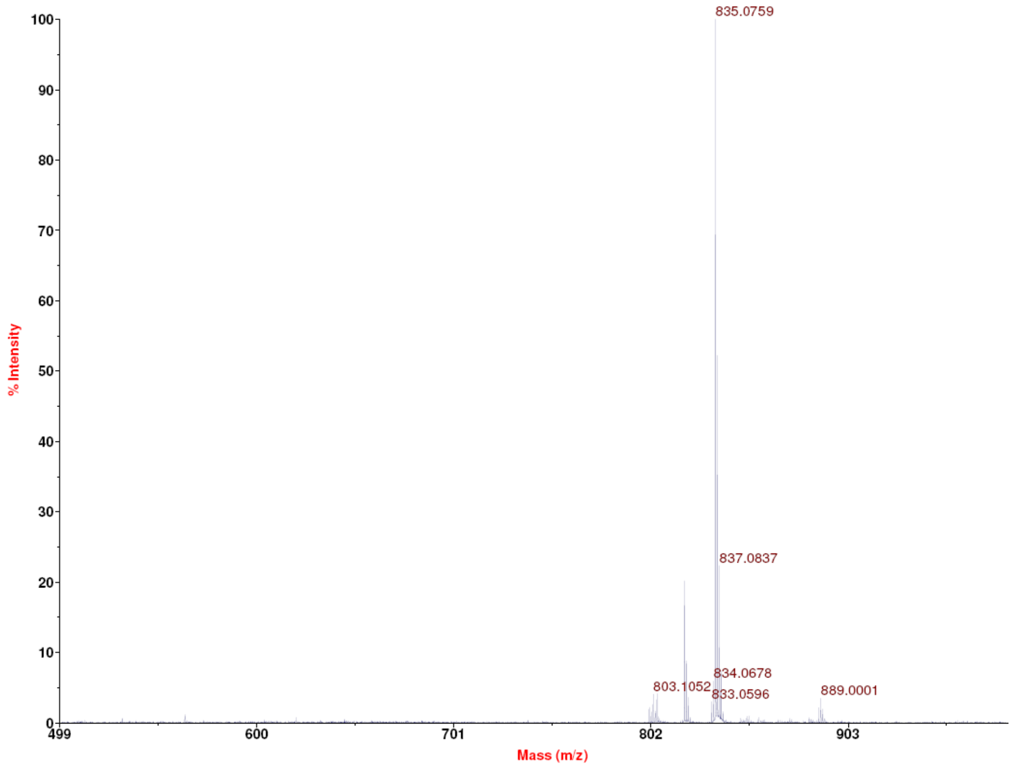

Figure S3. ^{1}H NMR spectrum and high-resolution mass spectrum of chromophore **2**

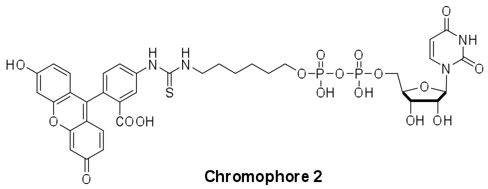

Chromophore 2

$C_{36}H_{37}N_4O_{17}P_2S$ $(M-H)^-$: calcd 891.1350, found 891.1348.

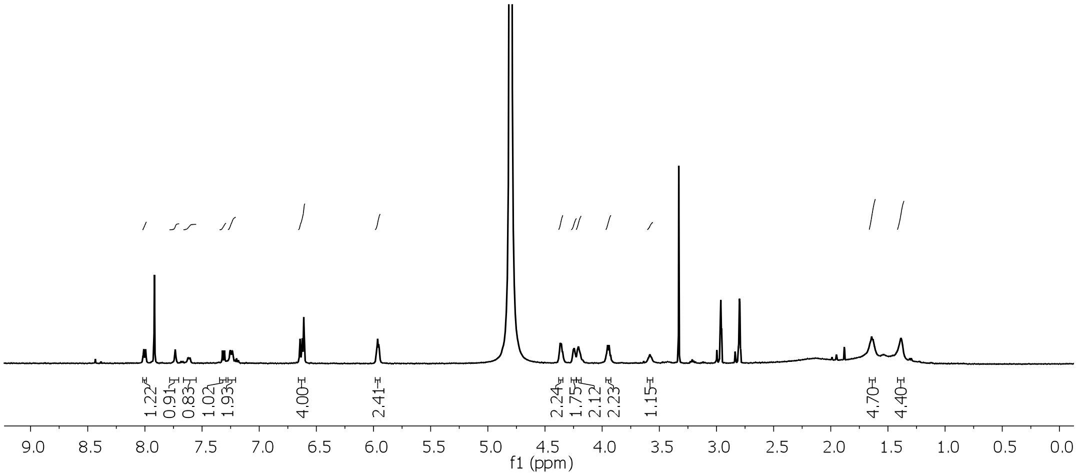

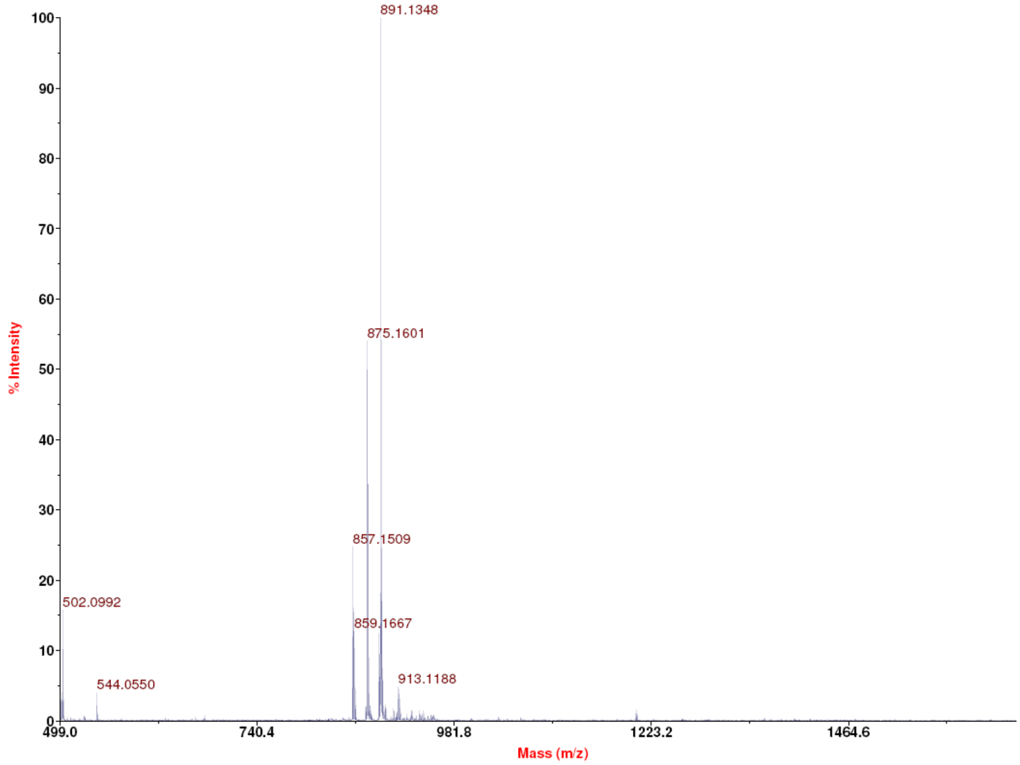

Figure S4. ^{1}H NMR spectrum and high-resolution mass spectrum of chromophore **3**

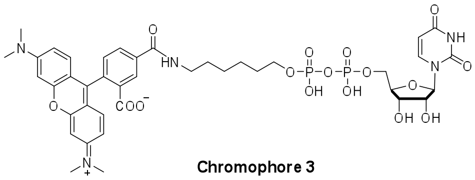

Chromophore 3

$C_{40}H_{46}N_5O_{16}P_2$ $(M\text{-}H)^-$: calcd 914.2415, found 914.2431.

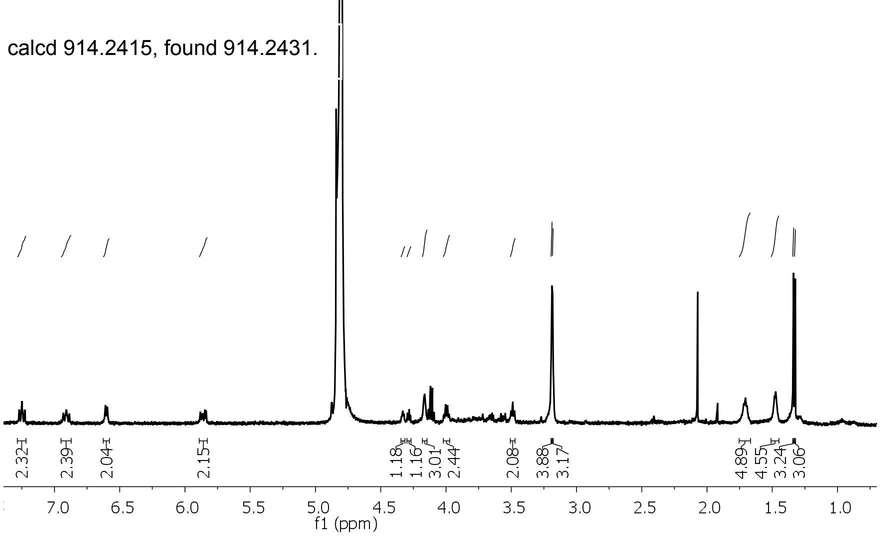

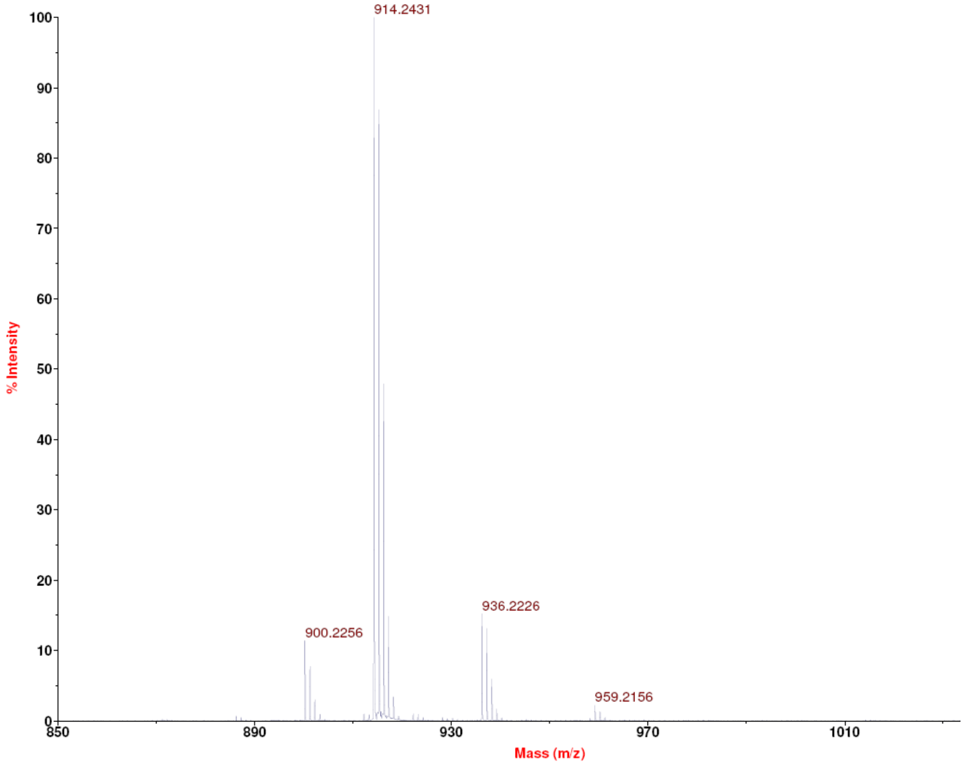

Figure S5. High-resolution mass spectrum of chromophore **2**

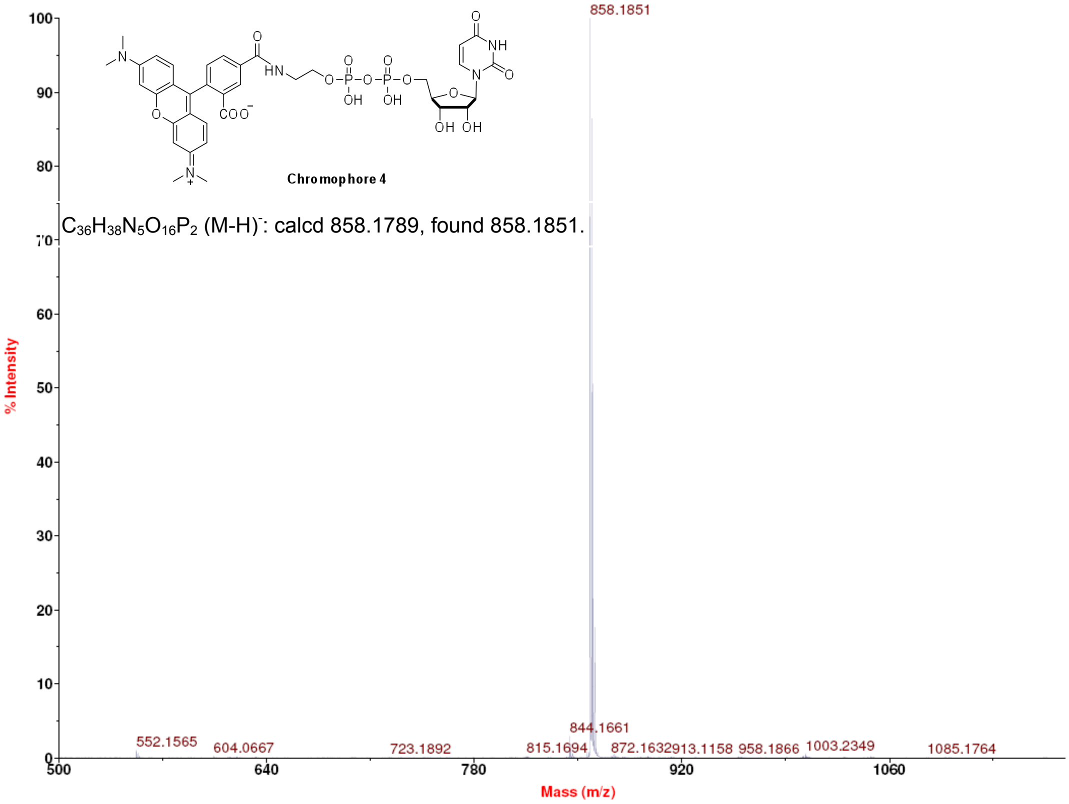

Supplement: Supplementary file 1 — The Supporting Material includes the UV-visible spectrum and SDS-PAGE of AfUGM, and 1H NMR and High-resolution MS spectra of the four UDP chromophores. UV-visible spectrum was obtained on Agilent photospectrometer. NMR spectral data were obtained using a JEOL Eclipse spectrometer at 500 MHz, or a Varian Inova spectrometer at 400 MHz. Chemical shifts were reported as σ-values relative to known solvent residue peaks. High-resolution mass spectra (HRMS) were obtained in the Mass Spec Incubator of Department of Biochemistry at Virginia Tech. [file 513905.f1.pdf]
